# Quiescin Sulfhydryl Oxidase 2 Overexpression Predicts Poor Prognosis and Tumor Progression in Patients With Colorectal Cancer: A Study Based on Data Mining and Clinical Verification

**DOI:** 10.3389/fcell.2021.678770

**Published:** 2021-11-10

**Authors:** Tao Jiang, Li Zheng, Xia Li, Jia Liu, Hu Song, Yixin Xu, Chenhua Dong, Lianyu Liu, Hongyu Wang, Shuai Wang, Renhao Wang, Jun Song

**Affiliations:** ^1^Department of General Surgery, The Affiliated Hospital of Xuzhou Medical University, Xuzhou, China; ^2^Institute of Digestive Diseases, Xuzhou Medical University, Xuzhou, China; ^3^State Key Laboratory of Proteomics, Beijing Proteome Research Center, National Center for Protein Sciences, Beijing Institute of Lifeomics, Beijing, China; ^4^State Key Laboratory of Food Nutrition and Safety, Tianjin University of Science and Technology, Tianjin, China; ^5^The Graduate School, Xuzhou Medical University, Xuzhou, China; ^6^Department of Pathology, The Affiliated Hospital of Xuzhou Medical University, Xuzhou, China; ^7^School of Life Sciences, Xuzhou Medical University, Xuzhou, China

**Keywords:** QSOX2, colorectal cancer, TCGA, TMAs, prognosis, GSEA

## Abstract

**Background:** As a member of the atypical thiol oxidase family, quiescin sulfhydryl oxidase 2 (QSOX2) has been reported to play an important role in several biological processes, but the expression and function of QSOX2 in colorectal cancer (CRC) remains elusive.

**Methods:** The difference of QSOX2 expression, and its relationship with clinicopathological features and prognosis in CRC, was analyzed by bioinformatic analysis and validated by clinical CRC specimen cohort. The functional characterization of QSOX2 was detected via *in vitro* and *vivo* experiments in CRC cell lines, while the potential signaling pathways were predicted by Gene Set Enrichment Analysis (GSEA).

**Results:** Our data based on bioinformatical analysis and clinical validation demonstrated that the expression of QSOX2 in CRC tissues was significantly upregulated. Additionally, the chi-square test, logistic regression analysis, and Fisher’s exact test showed that QSOX2 overexpression was significantly correlated with advanced clinicopathological parameters, such as pathological stage and lymph node metastasis. The Kaplan–Meier curves and univariate Cox regression model showed that QSOX2 overexpression predicts poor overall survival (OS) and disease-free survival (DFS) in CRC patients. More importantly, multivariate Cox regression model showed that QSOX2 overexpression could serve as an independent factor for CRC patients. *In vitro* and *vivo* data showed that the proliferation and metastasis ability of CRC cells were suppressed on condition of QSOX2 inhibition. In addition, GSEA showed that the QSOX2 high expression phenotype has enriched multiple potential cancer-related signaling pathways.

**Conclusion:** QSOX2 overexpression is strongly associated with malignant progression and poor oncological outcomes in CRC. QSOX2 might act as a novel biomarker for prognosis prediction and a new target for biotherapy in CRC.

## Introduction

Colorectal cancer, including colon cancer and rectal cancer, has leaped from the third leading cause of cancer death to the second cause in the world, which poses a more serious threat to the health of the people (Siegel et al., 2021). In the current clinical treatment, the standard therapy for CRC is surgery followed by chemotherapy or radiation (Provenzale et al., 2020). Due to the heterogeneity of CRC, patients may exhibit varied responses to the standard therapy (Zhai et al., 2017). Multistep and multifactorial process involved in several genetic alterations and biological pathway activation or inhibition may contribute to explain the mechanism of colorectal carcinogenesis and progression (Nguyen et al., 2020). In the past two decades, next-generation genome sequencing technology and numerous reports have identified some effective biomarkers, such as KRAS, SMAD2, BRAF, and PIK3CA/PIK3CB, which can assist doctors to predict the effect of adjuvant chemotherapy and the prognosis and recurrence of patients (De Roock et al., 2010; Sepulveda et al., 2017). Nevertheless, these findings are far from enough; more powerful and reliable biomarkers needs to be discovered, which will help doctors to develop new methods to improve clinical efficacy of CRC patients (Koncina et al., 2020).

Genes that contribute to oncogenicity has the potential to be explored for identifying cancer biomarkers and discovering new drug targets (Kamel and Al-Amodi, 2017). The atypical thiol oxidase family including two classes of QSOX transcript variants in vertebrates comprising, namely, QSOX1 and QSOX2, respectively (Codding et al., 2012; Israel et al., 2014). As the most wildly studied type, QSOX1 was observed to be upregulated and promote progression in multiple cancers, including prostate cancer, breast cancer, glioblastoma, lung cancer, pancreatic cancer, and malignant pleural mesothelioma (Katchman et al., 2011; Araujo et al., 2014; Knutsvik et al., 2016; Baek et al., 2018; Sung et al., 2018; Geng et al., 2020; Lacerenza et al., 2020). It is worth noting that upregulated QSOX1 expression was associated with features of poor outcomes in breast cancer, such as HER2 positivity and hormone receptor negativity (Knutsvik et al., 2016). As a highly homologous paralog of QSOX1, the human QSOX2 gene, chromosome 9q34.3, comprises 12 exons and encodes a putative protein of 698 amino acids (Hoober et al., 1999). A previous study has reported that the expression of QSOX2 appears to be lower than QSOX1 in most human normal tissues, such as the ovaries, prostate, and lung tissues (Coppock and Thorpe, 2006). Nevertheless, the exact expression and biological properties of QSOX2 in cancer remains to be elucidated. Given that QSOX2 and QSOX1 have 40% identity in the primary amino acid structure and higher identity in the functional domain, reaching 68% (Wang et al., 2018), we suspected that QSOX2 might also participate in the tumorigenesis and progression of cancers. However, the detailed expression status, prognostic role, and biological function of QSOX2 in human cancers, including CRC, remain unknown.

In this study, we first evaluate the mRNA and protein expression and asses the prognostic significance of QSXO2 in CRC based on bioinformatic analysis and clinical traits. In addition, experiments *in vitro* and *in vivo* were conducted to detect the biological function of QSOX2 in cell proliferation and metastasis of CRC. Furthermore, the QSOX2-related signaling pathways involved in CRC, which may support further investigation into the molecular mechanism underlying CRC, were analyzed by GSEA and verified by GEPIA correlation analysis and Western blotting analysis. Our results demonstrated that QSOX2 can be a promising biomarker for prognosis prediction in CRC.

## Materials and Methods

### Publicly Available Database Analysis

Online public available databases, UALCAN^1^ and TIMER2.0,^2^ were used to observe the expression of QSOX2 in human pan-cancer in mRNA and protein level (Chandrashekar et al., 2017; Li et al., 2020). The correlation of mRNA level between QSOX2 and signaling pathway-related genes was analyzed by the online database Gene Expression Profiling Interactive Analysis (GEPIA)^3^ (Tang et al., 2017).

### Bioinformatics Analysis Based on TCGA Cohort

The original mRNA expression data of patients with COAD or READ, followed by corresponding basic information, such as gender, age, etc., and clinical information, such as tumor staging, etc., were downloaded from TCGA official website.^4^ In total, the data of 452 COAD tissues, 41 adjacent normal tissues, 6 READ tissues, and 1 adjacent normal tissue were downloaded. The details of the COAD and READ patients are shown in Table 1. For further bioinformatic analysis, 314 COAD and READ cases were involved; those cases with expression or characteristic data that were incomplete and biased were excluded from further bioinformatic analysis. Then the original gene expression data were sorted and merged by the Perl programming language, while the QSOX2 expression was extracted by the limma package of R software. The extracted data were visualized by the limma package, while scatter difference diagrams were drawn by the beeswarm package. We use the R software to visualize the analysis results.

**TABLE 1:** Characteristics of patients with colorectal cancer (CRC) [colon adenocarcinoma (COAD) and rectum adenocarcinoma (READ)] in TCGA database.

Characteristics	Variable	Patients (458)	Percentages (%)
Age	<65 years	170	37.12
	≥65 years	288	62.88
Gender	Male	241	52.62
	Female	217	47.38
Pathological stage	I	78	17.03
	II	180	39.30
	III	125	27.29
	IV	64	13.97
	Unknown	11	2.40
T classification	Tis	1	0.22
	T1	11	2.40
	T2	78	17.03
	T3	311	67.90
	T4	57	12.45
N classification	N0	273	59.61
	N1	104	22.71
	N2	81	17.69
M classification	M0	338	73.80
	M1	64	13.97
	MX	49	10.70
	Unknown	7	1.53
Vital status	Alive	369	80.57
	Death	89	19.43

*Data are presented as number (%).*

### Verification of Quiescin Sulfhydryl Oxidase 2 Expression by the Gene Expression Omnibus Database

A search of RNA sequencing and the microarray data, which met the requirements from the Gene Expression Omnibus (GEO) dataset, was conducted, and a total of seven datasets (GSE106582, GSE37182, GSE83889, GSE21815, GSE21510, GSE9348, and GSE35279) were obtained, including 291 adjacent non-tumor tissues (adjacent NTTs), 661 CRC tissues, and original QSOX2 mRNA expression data (Table 2). The differences in QSOX2 expression were detected by the comprehensive meta-analysis via Review Manage 5.3. The combined value was calculated by standard mean difference (SMD) with a 95% confidence interval (CI). χ^2^ and *I*^2^ statistical tests were used to evaluate the heterogeneity between the included datasets. In condition of *p* > 0.05 or *I*^2^ < 50%, the fixed effect model was used for calculating the combined effect; otherwise (*p* < 0.05 or *I*^2^ > 50%), the random effect model was used. The results are visualized by forest plots.

**TABLE 2:** Information of GEO dataset involved in this study.

GEO dataset	Time	Country	GPL	Sample	N
GSE106582	2018	Germany	GPL10558	N	117
				T	77
GSE37182	2018	Italy	GPL6947	N	88
				T	84
GSE83889	2018	South Korea	GPL10558	N	35
				T	101
GSE21815	2019	Japan	GPL6480	N	9
				T	132
GSE21510	2019	Japan	GPL570	N	25
				T	123
GSE9348	2019	Singapore	GPL570	N	12
				T	70
GSE35279	2019	Japan	GPL6480	N	5
				T	74

*GEO, Gene Expression Omnibus; N, normal; T, tumor; GPL, GEO platform.*

### Paraffin-Embedded and Fresh Frozen Tissue Specimen Cohort

A cohort of 568 formalin-fixed paraffin-embedded CRC cases was collected to conduct the tissue microarrays (TMAs) for IHC analysis. Details about the TMAs has been previously described (Jiang et al., 2018). The other cohort consists of 50 pairs of fresh frozen CRC tumor and adjacent NTTs ranging from September 2020 to January 2021 were collected at the same hospital and preserved at −80°C for qRT-PCR. The Ethics Committee of the Affiliated Hospital of Xuzhou Medical University approved the project, and the patients or their family members signed the informed consent form.

### Immunohistochemistry Staining and Evaluation

IHC staining was performed on TMAs consisting of 568 formalin-fixed paraffin-embedded sections following a standard streptavidin–peroxidase (SP) method that has been previously described (Hou et al., 2018). Anti-QSOX2 antibodies were applied with 1:200 dilutions for primary antibody incubation (HPA012716, Sigma-Prestige, United States). The images of TMAs staining were obtained by an Olympus microscope (Tokyo, Japan).

The evaluation of the immunohistochemical reaction product has been previously described (Bai et al., 2019). The staining of QSOX2 was assessed blindly and independently by two pathologists, and all disagreements will be resolved through the participation of a third pathologist. Positive QSOX2 immunostaining signal was observed predominantly in the cytoplasm. The signals were quantified based on the intensity and percentage of positively stained cells. The QSOX2 staining intensity was scored 0–3 (0 = negative; 1 = weak; 2 = moderate; 3 = strong). The proportion of QSOX2-positive stained cells was scored as 1 (0–25%), 2 (26–50%), 3 (51–75%), and 4 (76–100%). The level of QSOX2 staining was evaluated by immunoreactivity score (IRS), which is calculated by multiplying the scores of staining intensity and percentage. According to IRS, the QSOX2 staining pattern was categorized as negative (IRS: 0), weak (IRS: 1–3), moderate (IRS: 4–6), and strong (IRS: 8–12). QSOX2 expression was classified as low (IRS: 0–4) or high (IRS: 6–12) group.

### RNA Extraction and Quantitative Real-Time PCR

Trizol reagent (Invitrogen) was used to extract the total RNA from fresh frozen CRC tumor and adjacent NCTs according to the instructions of the manufacturer, and then the Trans Script one-step guide DNA removal and complementary DNA synthesis super mix (Trans Gen Biotech) was used for the reverse transcription reaction. The sequences of all the primers amplified for quantitative real-time PCR (qRT-PCR) were listed after: 5′-TCCCTTCTTGACAACCGTGG-3′ (forward) and 5′-AAATGCTTTGTCCCCGTCCA-3′ (reverse) for QSOX2; 5′-TTTCTCTCGGCTCCCCATGT-3′ (forward) and 5′-GCTGTATATTCAGCATTGTGGG-3′ (reverse) for p21; 5′-ACGGGAGCCCTAGCCTGGAGC-3′ (forward) and 5′-TGCCCTTCTCCACCTCTTGCC-3′ (reverse) for p27; 5′-TGAGGGACGCTTTGTCTGTC-3′ (forward) and 5′-CTTCTGCTGGAAACATGCCG-3′ (reverse) for cyclin D1; 5′-TAGCTGGTCTGGCGAGGTTT-3′ (forward) and 5′-ACAGGTGGCCAACAATTCCT-3′ (reverse) for cyclin E2; 5′-AAGGTCGGAGTCAACGGATTTG-3′ (forward) and 5′-CCATGGGTGGAATCATATTGGAA-3′ (reverse) for GAPDH; 5′-ATGGAGAACTTCCAAAAGGTGG-3′ (forward) and 5′-TCAGAGTCGAAGATGGGGTAC-3′ (reverse) for CDK2; 5′-ATGGCTACCTCTCGATATGAGC-3′ (forward) and 5′-CATTGGGGACTCTCACACTCT-3′ (reverse) for CDK4.

### Colorectal Cancer Cell Culture and Transfection

HCT116 and LoVo, two types of human CRC cell lines, were gained from the Chinese Academy of Sciences Cell Bank. HCT116 and LoVo were cultured in DMEM Medium and RPMI 1640 medium, respectively, both supplemented with 10% fetal bovine serum (FBS) (Gibco, United States). All cell lines were incubated in a 37°C humidified incubator with 5% CO_2_. The small interfering RNAs (siRNAs) against human QSOX2 and non-specific siRNA used as negative controls were gained from Gene pharma Technology (Shanghai, China) and transfected into the CRC cells by SilenFect reagent (Bio-Rad Laboratories, Inc.). All the experiments were performed according to the protocol of the manufacturer. The sequences of siRNAs were described as follows: siQSOX2#1 sense: GCAGCCAUUACGUGGCUAUTT; siQSOX2#2 sense: GGUACGUUCACACCUUCUUTT; siCtrl sense: UUCUCCGAACGUGUCACGUTT.

### Transwell and Wounding Assay

Transwell filter inserts with a pore size of 8 μm (8.0 μm, Corning, NY, United States) were used to detect cell migration and invasion, which were carried out using invasion assay. The modified two-chamber plates were coated with Matrigel (BD Biosciences, Mississauga, Canada), while in the migration assay, they was not. After serum starvation overnight, cells were seeded in the top chamber accompanied by complete medium added to the bottom chambers. After incubation at 37°C with 5% CO_2_ for 24–48 h, the migration and invasion cells were fixed and stained before calculation. In wound healing assay, CRC cells at a density of 1 × 10^6^ cell/well were seeded onto six-well plates and cultured to a density of about 80%. Then artificial scratches were formed by sterile 10-μl pipette tips for each well. The suspended cells were cultured in a medium with 1% FBS after washing away with PBS. An inverted light microscope (IX71; Olympus, Tokyo, Japan) was used to photograph cell migration distance at 0 and 48 h.

### Cell Counting Kit-8 and 5-Ethynyl-2′-Deoxyuridine Assay

HCT116 and LoVo cells were transfected with Ctrl siRNA or QSOX2 siRNA. For cell counting kit (CCK)-8 assay, the cells were counted before laying in a 96-well plate with 2,000 cells per well and continued to be cultured at 37°C in 5% CO_2_ for 0, 24, 48, and 72 h. At specified time-points, the cells in each well will be added to 10 μl of cell counting kit (CCK)-8 solutions (Dojindo Molecular Technologies, Inc., Kumamoto, Japan) and continued to be cultured under the original conditions for 2 h. Absorbance was measured at 450 nm at the specified time-points. All experiments were performed in triplicate. For EdU (5-ethynyl-2′-deoxyuridine) assay, the cells were laid in a 96-well plate with 4,000 cells per well and cultured at 37°C in 5% CO_2_ for 20 h. Then, the cells were treated with 50 μmol/L of EdU assay kit (RiboBio, Guangzhou, China) according to the protocol of the manufacturer and incubated at 37°C in 5% CO_2_ for 2 h. Finally, 4% paraformaldehyde was used to fix the cells for 20 min, 0.5% Triton X-100 was used to permeabilize the cells for another 20 min, 100 μl of 1 × Apollo reaction cocktail was used to incubate the cells for 30 min at room temperature.

### Gene Set Enrichment Analysis

As a computational method, the Gene Set Enrichment Analysis (GSEA) was performed to analyze the statistically significant and concordant differences between two different biological states based on *a priori* set of genes (Subramanian et al., 2007). According to the median value of QSOX2 expression, the CRC samples obtained from TCGA dataset were divided into the QSXO2 high and low groups, respectively. “c2.cp.kegg.v7.0.symbols.gmt,” which was an annotated gene set, was finally selected as the reference gene set. In order to identify the significantly different signaling pathways, 1,000 times was set for analyzing the number of gene set permutations each time. The statistical significance and importance of the correlation between gene sets and signaling pathways were determined by nominal *p*-value, normalized enrichment score (NES), and false discovery rate (FDR) *q*-value.

### Western Blot Analysis

Proteins were extracted from HCT116 and LoVo cells with RIPA buffer (Beyotime, Shanghai, China) supplemented with protease inhibitor cocktail and quantified by bicinchoninic acid (BCA) analysis (Beyotime, Shanghai, China). The prepared protein was loaded into a 10% SDS-PAGE gel with a content of 30 μg per well, then transferred to PVDF membranes, and blocked with 5% skimmed milk. Then, the PVDF membranes were incubated at 4° with antibodies against QSOX2 (1:1,000, Abcam, MA, United States), p21 (1:1,000, Cell Signaling Technology, MA, United States), p27 (1:1,000, Cell Signaling Technology, MA, United States), Cyclin D1 (1:1,000, Cell Signaling Technology, MA, United States), Notch1 (1:1,000, Cell Signaling Technology, MA, United States), GAPDH (1:5,000, Proteintech, Wuhan, China) for one night. The next day, the PVDF membranes were washed with TBST three times and then incubated with the secondary antibody for 2 h at room temperature. Finally, ECL substrate (Labgic Technology Co., Ltd., Beijing, China) was used to visualize the blots.

### Xenograft Tumorigenesis Study

The ethical approval of animal experiments was approved by the Animal Care and Use Committee of Xuzhou Medical University. Four to 6-week-old BALB/c nude mice (female) were obtained from the Vital River Laboratory Animal Technology Co., Ltd. (Beijing, China) and housed under specific pathogen-free conditions. The lentiviral against human QSOX2 (shQSOX2 group) and non-specific lentiviral (shCtrl group) were transfected into HCT116 cells according to the instructions of the manufacturer. The stable HCT116 cell line including shCtrl and shQSOX2 groups were selected with puromycin and verified by Western blot. In order to construct models of xenograft tumor, 200 μl of serum-free DMEM containing groups of HCT116- shCtrl and HCT116–shQSOX2 cells (5 × 10^6^) were injected subcutaneously into the flanks of mice. From the fifth day after the injection of cells, tumor volume (V) was calculated every 3 days by the following formula: V = (long axis × short axis^2^)/2. The mice were sacrificed at 24 days, and the tumors were used for HE and IHC staining after dissected and weighted.

### Statistical Analysis

The difference in QSOX2 expression between non-paired cases was tested by Wilcoxon rank-sum test, while paired cases was tested by Wilcoxon matched-pairs signed-rank test. Kruskal–Wallis test was used to analyze the differences in QSOX2 expression among multiple clinical characteristic groups. Chi-square (χ^2^) test was used to analyze the relationship between QSOX2 expression and clinicopathological parameters. The survival rate differences between QSOX2 high and low groups, including OS and DFS rates, was analyzed by Kaplan–Meier analysis and log rank test. The univariate Cox proportional hazard regression model combined with multivariate Cox proportional hazard regression model were used for determining the effects of QSOX2 or other clinicopathological parameters on survival. SPSS statistical software (version 23.0) and R software (version 2.15.3) were used for statistical analyses. For all tests, *p* < 0.05 was defined as statistically significance.

## Results

### Quiescin Sulfhydryl Oxidase 2 Is Upregulated in Pan-Cancer and Colorectal Cancer

**FIGURE 1:**
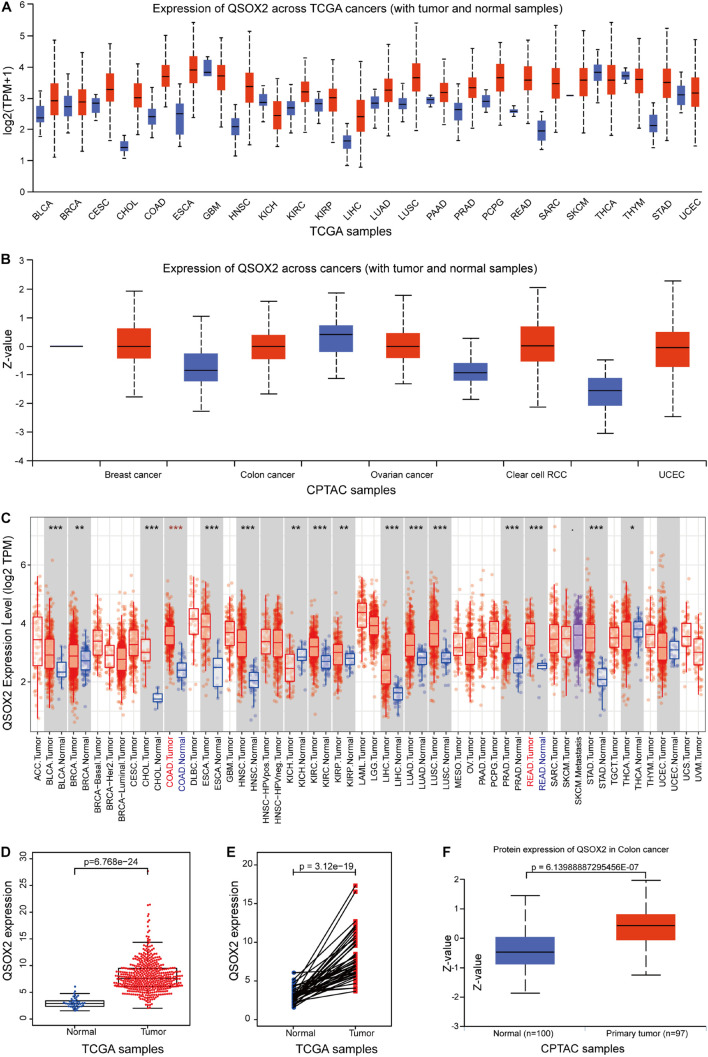
Quiescin sulfhydryl oxidase 2 (QSOX2) expression in pan-cancer and in colorectal cancer (CRC). **(A,B)** The QSOX2 expression of mRNA level in multiple human cancers and corresponding normal tissues in the UALCAN database. **(C)** The QSOX2 expression of mRNA level in different human cancers and corresponding normal tissues in the TIMER database (*p < 0.05; **p < 0.01; ***p < 0.001). **(D)** The QSOX2 expression of mRNA level in CRC tumors and corresponding normal tissues from TCGA database. **(E)** QSOX2 mRNA expression in pairs of tumor and normal tissues of CRC patients from TCGA database. **(F)** QSOX2 protein expression in the primary tumors and normal tissues of CRC patients from CPTAC database.

Given that QSOX2 has been poorly studied in human cancers, it is important to investigate whether QSOX2 is involved in human cancers. First, we analyzed the expression of QSOX2 in various cancer types using the public gene expression data available through TCGA and CPTAC. According to UALCAN analysis, compared with the corresponding normal tissue, the mRNA expression of QSOX2 was significantly upregulated in most human cancers (Figures 1A,B). Consistent with the analysis results of the UALCAN database, QSOX2 was also found to be upregulated in numerous human solid cancers according to the TIMER 2.0 online database, including COAD (colon adenocarcinoma) and READ (rectum adenocarcinoma) (Figure 1C). For further analysis, TCGA database was used to analyze the comprehensive expression of QSOX2 in CRC. The QSOX2 mRNA expression level in CRC tissues was significantly higher than that in the normal tissues (Figure 1D). Data from 41 tumors and paired adjacent NTTs of CRC patients in the TCGA database further corroborated these results (Figure 1E). The data from CPTAC database containing 97 CRC patients and 100 adjacent NTTs validated further the result of TCGA database (Figure 1F).

### Validation of Quiescin Sulfhydryl Oxidase 2 Upregulation in Colorectal Cancer by Tissue Microarrays, Quantitative Real-Time PCR, and Standard Mean Difference

**FIGURE 2:**
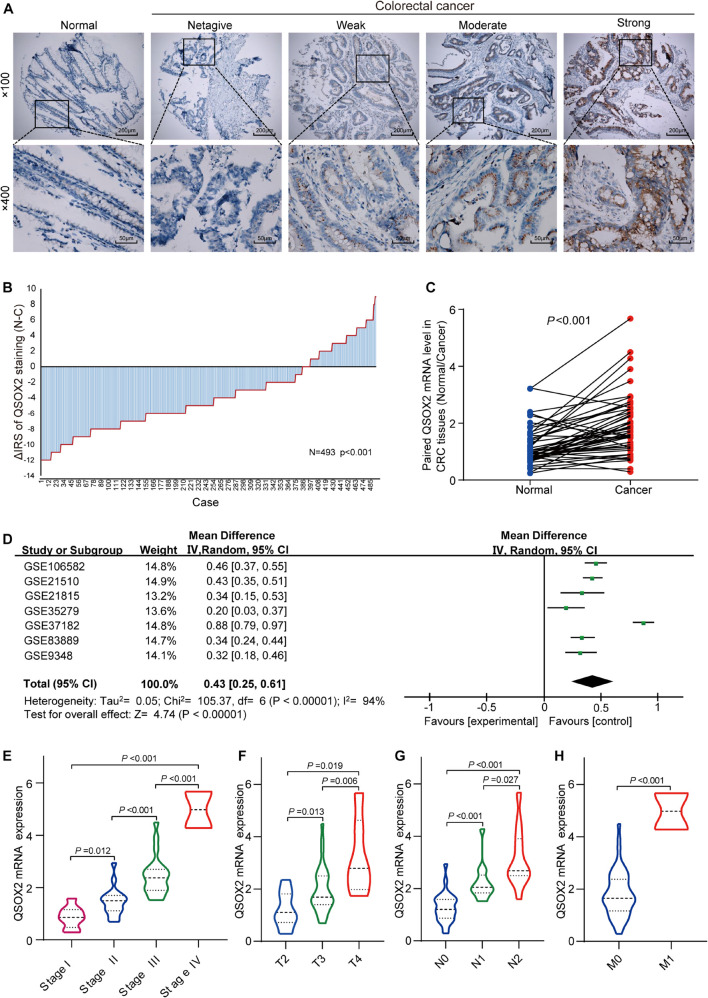
Expression of QSOX2 is upregulated in clinical CRC patient specimens and GEO microarrays. **(A)** Representative images of QSOX2 expression in the tumor microarrays (TMAs) using immunohistochemistry (IHC) staining. **(B)** QSOX2 protein expression was significantly upregulated in cancerous tissues (*p* < 0.001, the paired Wilcoxon test). Note: N, paired adjacent non-tumor tissues (NTTs); C, CRC tissues. **(C)** The mRNA expression of QSOX2 in 50 cancerous tissues and corresponding non-tumor tissues were detected by quantitative real-time PCR (qRT-PCR) (*p* < 0.001). **(D)** Forest plot of QSOX2 expression data from GEO microarrays. Note: GEO, Gene Expression Omnibus; SMD, standard mean difference; CI, confidence interval. **(E–H)** Violin plot evaluating QSOX2 mRNA expression in different groups classified according to clinical characteristics. **(E)** TNM stage. **(F)** Depth of invasion. **(G)** Lymph node metastasis. **(H)** Distant metastasis.

To characterize QSOX2 expression status in CRC, we first evaluated the endogenous QSOX2 expression in 568 CRC samples and adjacent NTTs in the TMAs using immunohistochemistry (IHC) (Figure 2A). Finally, 493 CRC specimens were included in the analysis, and the remaining samples were lost due to antigen retrieval. The paired Wilcoxon test showed that QSOX2 protein expression was significantly upregulated in cancerous tissues (*p* < 0.001) (Figure 2B). To further verify the difference in QSOX2 expression in the TCGA database, QSOX2 mRNA expression was validated in a small CRC cohort containing 50 pairs of fresh frozen tissue specimens. The results of qRT-PCR showed that that QSOX2 mRNA expression level was also upregulated in cancerous tissues relative to adjacent NTTs (*p* < 0.001) (Figure 2C). Additionally, the differences of QSOX2 expression in CRC were detected by the comprehensive meta-analysis in the GEO dataset (Table 2). The data showed that the I-square value was 94% (*p* < 0.001), according to the random effects model (95% CI:0.25–0.61), the final combined SMD of QSOX2 was 0.43. The data are statistically significant, thus, suggesting that QSOX2 expression was upregulated in CRC based on the GEO database (Figure 2D).

### Quiescin Sulfhydryl Oxidase 2 Is Associated With Malignant Progression in Patients With Colorectal Cancer From TCGA Cohort

As we continue to explore the mRNA expression of QSOX2 in TCGA database, we were surprised to find that the expression of QSOX2 was different in groups classified by tumor pathological stage (*p* = 0.004), T classification (*p* < 0.001), N classification (*p* =0.009), and M classification (*p* < 0.001) (Supplementary Figures 1A–D). To further analyze the relationship between QSOX2 expression and clinicopathological features, we collected original expression information and corresponding clinical data of 314 patients from TCGA database. As shown in Table 3, QSOX2 high expression was positively associated with pathological stage progression (*p* = 0.003) and lymph node metastasis (*p* = 0.003). Logistic regression analysis indicated that the QSOX2 overexpression was observed to be positively correlated with pathological stage (stage II vs. stage I, OR = 2.211, *p* = 0.007; stage III vs. stage I, OR = 5.049, *p* < 0.001; stage IV vs. stage I, OR = 3.088, *p* = 0.007), lymph node metastasis (N1/N2 vs. N0, OR = 2.239, *p* = 0.001) (Table 4).

**TABLE 3:** The relationship between quiescin sulfhydryl oxidase 2 (QSOX2) expression and CRC clinicopathological features based on TCGA cohort.

Variables	QSOX2 expression		Total	*p*-value
	High (*n* = 224)	Low (*n* = 90)		
**Age**				
<65 years	80 (70.2)	34 (29.8)	114	0.731
≥65 years	144 (72.0)	56 (28.0)	200	
**Gender**				
Male	119 (73.0)	44 (27.0)	163	0.497
Female	105 (69.5)	46 (30.5)	151	
**Pathological stage**				
I and II	96 (63.6)	55 (36.4)	151	0.003
III and IV	128 (78.5)	35 (21.5)	163	
**T classification**				
T1 and T2	34 (66.7)	17 (33.3)	51	0.420
T3 and T4	190 (72.2)	73 (27.8)	263	
**Lymph node metastasis**				
N0	103 (64.8)	56 (35.2)	159	0.003
N +	131 (79.4)	34 (20.6)	165	
**Distant metastasis**				
M0	176 (69.6)	77 (30.4)	253	0.157
M +	48 (78.7)	13 (21.3)	61	

**TABLE 4:** QSOX2 expression correlated with clinicopathological features based on TCGA cohort.

Clinicopathological features	Total (*N*)	Odds ratio in QSOX2 expression	*p*-value
**Age**			
<65 vs. ≥ 65	447	1.135 (0.774–1,667)	0.517
**Gender**			
Male vs. female	447	0.829 (0.571–1.201)	0.321
**Pathological stage**			
Stage II vs. stage I	131	2.211 (1.077–4.654)	0.007
Stage III vs. stage I	129	5.049 (2.418–10.946)	0.000
Stage IV vs. stage I	104	3.088 (1.383–7.093)	0.007
**T classification**			
T2 vs. T1	59	1.500 (0.231–12.078)	0.670
T3 vs. T1	196	1.454 (0.236–11.221)	0.686
T4 vs. T1	50	1.714 (0.260–13.974)	0.575
**Lymph node metastasis**			
Yes vs. no	272	2.239 (1.382–3.654)	0.001
**Distant metastasis**			
Yes vs. no	367	1.226 (0.707–2.138)	0.469

### High Quiescin Sulfhydryl Oxidase 2 Expression Is Associated With Advanced Clinicopathological Parameters in Patients With Colorectal Cancer During the Clinical Validation Phase

Consistent with the analysis results of TCGA cohort, QSOX2 mRNA expression in the CRC cohort containing 50 pairs of fresh frozen tissue specimens was also different in groups classified according to TNM stage, lymph node metastasis, depth of invasion, and distant metastasis (Figures 2E–H). To further understand the clinical significance of QSOX2 in the large CRC cohort containing 568 formalin-fixed paraffin-embedded CRC specimens, Fisher’s exact test was used to analyze the relationship between QSOX2 expression and clinicopathological features. Table 5 showed the relationship between QSOX2 expression and clinicopathological features of the CRC patients. QSOX2 overexpression was positively associated with aggressive features of CRC, such as tumor lymph node metastasis (*p* = 0.048), TNM stage progression (*p* < 0.001), tumor diameter (*p* = 0.017), and distant metastasis (*p* = 0.008). On the other hand, there was no striking relationship between QSOX2 expression and age, gender, differentiation, or depth of invasion.

**TABLE 5:** Relationship between QSOX2 expression and clinicopathological features of CRC patients based on clinical CRC specimen cohort.

Variables	Cases	QSOX2 expression (*n* = 493 cases)		*p*-value
		Low	High	
All patients	493	164	329	
**Age (years)^b^**		62 ± 12	61 ± 13	0.531
≤60	211	72	139	
>60	282	92	190	
**Gender^a^**				0.923
Males	277	93	184	
Females	216	71	145	
**Lymph node metastasis^a^**				0.048
N0	309	113	196	
N1/N2/N3	184	51	133	
**TNM stage^a^**				<0.001
I/II	271	116	155	
III/IV	222	48	174	
**Differentiation^a,c^**				0.18
Poor	93	37	56	
Moderate/high	395	127	268	
**Tumor diameter^a^**				0.017
≤5 cm	260	99	161	
>5 cm	233	65	168	
**Distant metastasis^a^**				0.008
M0	433	153	280	
M1	60	11	49	
**Depth of invasion^a^**				0.663
T1/T2	128	45	83	
T3/T4	365	119	246	

*^a^Two-sided Fisher’s exact tests.*

*^b^Two-sided Student’s t-test.*

*^c^The type of differentiation of cancer in five patients cannot be assessed.*

### High Quiescin Sulfhydryl Oxidase 2 Expression Is Related to Poor Survival in Patients With Colorectal Cancer Based on TCGA Cohort

**FIGURE 3:**
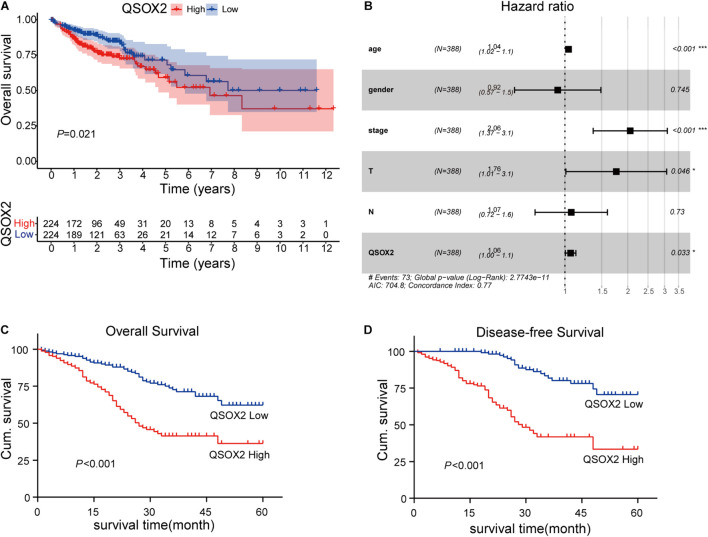
Prognostic role of QSOX2 in CRC patients. **(A)** Kaplan–Meier survival curves show patients of CRC with high QSOX2 expression exhibited shorter overall survival (OS) (*n* = 224, *p* = 0.021) based on TCGA database. **(B)** Forest plot shows that QSOX2 expression was an independent risk factor for CRC based on TCGA data (HR, 1.06; 95% CI, 1.00–1.13; *p* = 0.033). HR, hazard ratio; CI, confidence interval; *p < 0.05; ***p < 0.001. **(C,D)** Kaplan–Meier survival curves show that patients of CRC with high QSOX2 expression exhibited shorter OS (*n* = 493, *p* < 0.001) and disease-free survival (DFS) based on clinical specimen cohort.

The analysis of QSOX2 prognostic signature in CRC based on TCGA cohort was investigated by Kaplan–Meier risk estimates. The result showed that QSOX2 overexpression was positively associated with poor overall survival (Figure 3A). Besides, univariate and multivariate Cox proportional hazard regression models were conducted to assess the influence of QSOX2 expression and clinicopathological parameters on survival in CRC patients based on TCGA cohort. Univariate analysis revealed that QSOX2 expression (HR, 1.08; 95% CI, 1.02–1.14; *p* = 0.006), age (HR, 1.03; 95% CI, 1.01–1.05; *p* = 0.008), pathological stage (HR, 2.26; 95% CI, 1.73–2.94; *p* < 0.001), T classification (HR, 2.85; 95% CI, 1.80–4.52; *p* < 0.001), N classification (HR, 2.02; 95% CI, 1.54–2.65; *p* < 0.001), and M classification (HR, 4.45; 95% CI, 2.74–7.21; *p* < 0.001) were important risk factors for survival (Table 6). In addition, multivariate analysis showed that QSOX2 expression (HR, 1.06; 95% CI, 1.00–1.13; *p* = 0.033) was an independent risk factor for survival (Figure 3B and Table 6).

**TABLE 6:** Univariate and multivariate Cox proportional hazards analysis of overall survival (OS) in CRC patients based on TCGA cohort.

Parameters	Univariate analysis		Multivariate analysis	
	HR (95% CI)	*p*-value	HR (95% CI)	*p*-value
QSOX2	1.08 (1.02–1.14)	0.006	1.06 (1.00–1.13)	0.033
Age	1.03 (1.01–1.05)	0.008	1.04 (1.02–1.06)	<0.001
Gender	1.11 (0.70–1.77)	0.653	0.92 (0.57–1.49)	0.745
Pathological stage	2.26 (1.73–2.94)	<0.001	2.06 (1.37–3.09)	<0.001
T	2.85 (1.80–4.52)	<0.001	1.76 (1.01–3.07)	0.046
N	2.02 (1.54–2.65)	<0.001	1.07 (0.72–1.60)	0.730
M	4.45 (2.74–7.21)	<0.001		

*HR, hazard ratio; CI, confidence interval.*

### Upregulated Quiescin Sulfhydryl Oxidase 2 Expression Is a Predictor of Poor Prognosis in Colorectal Cancer During the Clinical Validation Phase

Similar results were observed during the clinical validation phase based on the survival follow-up information of 493 patients. Kaplan–Meier survival curves showed that the QSOX2 overexpression was positively associated with poor OS (*p* < 0.001) and DFS (*p* < 0.001) (Figures 3C,D). To further evaluate the prognostic significance of QSOX2 expression in CRC patients, univariate and multivariate Cox regression models were conducted in the 493 CRC specimens. Univariate Cox regression analysis suggested that QSOX2 expression, age, gender, TNM stage, LNM, tumor diameter, and distant metastasis were the significant risk factors for OS, while QSOX2 expression, depth of invasion, and TNM stage were the significant risk factors for DFS (Table 7). Finally, multivariate analysis showed that QSOX2 expression could serve as an independent prognostic biomarker for OS (HR, 2.512; 95% CI, 1.730–3.647; *p* < 0.001) and DFS (HR, 4.493; 95% CI, 2.640–7.649; *p* < 0.001) in CRC patients (Table 8).

**TABLE 7:** Univariate Cox regression analysis on OS and disease-free survival (DFS) in CRC based on clinical specimen cohort.

Variables^a^	Overall survival		Disease-specific survival	
	HR (95% CI)	*p*-value	HR (95% CI)	*p*-value
QSOX2	2.866 (1.989–4.128)	<0.001	5.149 (3.069–8.636)	<0.001
Age	1.424 (1.031–1.965)	0.032	1.008 (0.991–1.025)	0.359
Gender	1.294 (0.949–1.765)	0.103	1.456 (0.965–2.197)	0.074
Differentiate	0.865 (0.593–1.262)	0.451	0.789 (0.551–1.129)	0.194
Depth of invasion	1.260 (0.866–1.834)	0.227	1.266 (1.033–1.552)	0.023
TNM stage	1.781 (1.305–2.432)	<0.001	2.224 (1.448–3.418)	<0.001
LNM	1.344 (0.982–1.839)	0.065	1.408 (0.857–2.313)	0.177
Tumor diameter	1.455 (1.065–1.987)	0.019	1.300 (0.927–1.822)	0.128
Distant metastasis	2.719 (1.703–4.343)	<0.001		

*HR, hazard ratio; CI, confidence interval; LNM, lymph node metastasis.*

*^a^QSOX2: low vs. high; age: ≤ 60 vs. >60; gender: male vs. female; differentiate: poor vs. moderate and high; depth of invasion: T1 and T2 vs. T3 and T4; TNM stage was ranked as I and II vs. III and IV; LNM, N0 vs. N+; tumor diameter: ≤ 5 vs. >5; distant metastasis: M0 vs. M+.*

**TABLE 8:** Multivariate Cox regression analysis on OS and DFS in CRC based on clinical specimen cohort.

Variables^a^	Overall survival		Disease-free survival	
	HR (95% CI)	*p*-value	HR (95% CI)	*p*-value
QSOX2	2.512 (1.730–3.647)	<0.001	4.493 (2.640–7.649)	<0.001
Age	1.423 (1.024–1.977)	0.036	1.677 (1.067–2.634)	0.025
Gender	1.357 (0.979–1.879)	0.067	1.301 (0.826–2.047)	0.256
Tumor diameter	1.352 (0.986–1.853)	0.061	1.244 (0.811–1.909)	0.318
TNM stage	1.667 (1.201–2.314)	0.002	1.955 (1.207–3.166)	0.006
Differentiate	0.752 (0.514–1.101)	0.143	0.846 (0.526–1.361)	0.491

*HR, hazard ratio; CI, confidence interval; LNM, lymph node metastasis.*

*^a^QSOX2: low vs. high; age: ≤ 60 vs. >60; gender: male vs. female; differentiate: poor vs. moderate and high; depth of invasion: T1 and T2 vs. T3 and T4; TNM stage was ranked as I and II vs. III and IV; LNM: N0 vs. N+; tumor diameter: ≤ 5 vs. >5; distant metastasis: M0 vs. M+.*

### Quiescin Sulfhydryl Oxidase 2 Promotes Colorectal Cell Proliferation, Migration, and Invasion *in vitro*

**FIGURE 4:**
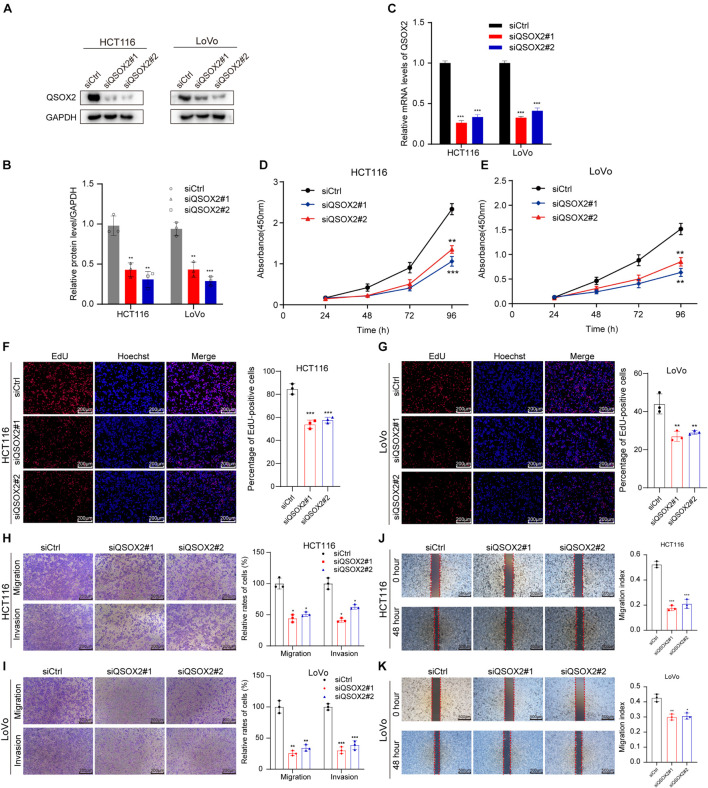
QSOX2 promotes CRC cell proliferation, migration, and invasion *in vitro*. **(A–C)** The inhibition of QSOX2 protein and mRNA expression was confirmed in HCT116 and LoVo cells by Western blotting and qRT-PCR, respectively. Densitometry values of QSOX2 normalized to GAPDH. **(D,E)** QSOX2 inhibition significantly reduced ability of proliferation in HCT116 and LoVo cells via cell counting kit 8 (CCK-8) assay. **(F,G)** QSOX2 inhibition significantly reduced ability of proliferation in HCT116 and LoVo cells via 5-ethynyl-2′-deoxyuridine (EdU) incorporation assay. **(H,I)** Inhibition of QSOX2 significantly suppressed the ability of migration and invasion in HCT116 and LoVo cells via Transwell assay. **(J,K)** Inhibition of QSOX2 significantly inhibited the wound healing speed in HCT116 and LoVo cells by wound healing assay. All experiments were repeated three times, and the final data are shown as mean ± standard deviations. *p < 0.05; **p < 0.01; ***p < 0.001.

Since the results of bioinformatic analysis and clinical specimens showed that QSOX2 overexpression was associated with the malignant progression of CRC patients, we will focus on the biological function of QSOX2 in CRC. The expression of QSOX2 was reduced by small interfering RNAs (siRNAs) and detected by Western blotting and qRT-PCR analysis, respectively (Figures 4A–C). Given that QSOX2 overexpression was positively associated with tumor diameter and lymph node metastasis, we wanted to detect whether QSOX2 facilitates the ability of proliferation and metastasis in CRC cells. CCK-8 and EdU assays showed that inhibition of QSOX2 significantly reduced rates of cell proliferation in HCT116 and LoVo cells (Figures 4D–G). Transwell migration and invasion assays, and wound healing assays were used to estimate the influence of QSOX2 expression on CRC metastasis. The Transwell assays showed that QSOX2 inhibition reduced the ability of migration and invasion in HCT116 and LoVo cells (Figures 4H,I). Wound healing assays showed that QSOX2 inhibition reduced the speed of wound healing in HCT116 and LoVo cells (Figures 4J,K).

### Identification of Quiescin Sulfhydryl Oxidase 2-Related Signaling Pathways by Gene Set Enrichment Analysis

**FIGURE 5:**
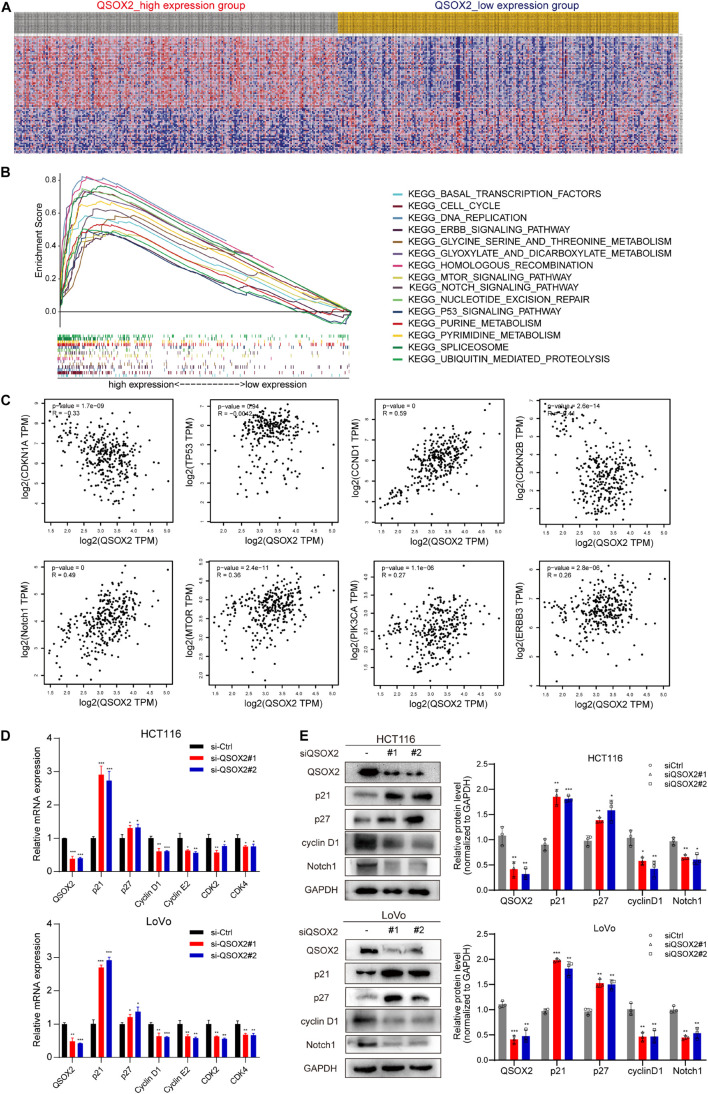
Mechanistic investigation into how QSOX2 promotes CRC progression. **(A)** The heat map generated based on genes enriched in QSOX2 high and low in TCGA database. **(B)** Enrichment plots from Gene Set Enrichment Analysis (GSEA) including enrichment score and gene sets. **(C)** Correlation between QSOX2 and signaling pathway-related genes, including p21, p27, cyclin D1, p53, Notch1, mTOR, PIK3CA, and ERBB3. **(D)** The e mRNA expression of cell cycle signaling pathway genes in CRC with knockdown of QSOX2 were determined by qRT-PCR. **(E)** The expression of QSOX2 and signaling pathway-related genes were detected by Western blotting, and GAPDH was used as a reference control. *p < 0.05; **p < 0.01; ***p < 0.001.

GSEA was conducted to detect the potential molecular mechanism and signaling pathways involved in how QSOX2 promotes CRC progression. First, TCGA samples were classified into two groups according to the expression of QSOX2. Then, the significance of multiple functional sets of QSOX2 high and low groups was analyzed by GSEA (Figure 5A). In the comprehensive analysis of NES, normal *p*-value, and FDR *q*-value, 15 signaling pathways enriched in the QSOX2 high group were selected, including cell cycle, homologous recombination, pyrimidine metabolism, spliceosome, purine metabolism, nucleotide excision repair, notch signaling pathway, glyoxylate, dicarboxylate metabolism, ubiquitin-mediated proteolysis, DNA replication, mTOR signaling pathway, p53 signaling pathway, ERBB signaling pathway, threonine metabolism, glycine serine, and basal transcription factors (Figure 5B and Table 9). In addition, correlation analysis by the GEPIA database showed that the mRNA level of p21, p27, cyclin D1, Notch1, mTOR, PIK3CA, and ERBB3 were significantly associated with QSOX2, while p53 was not, indicating that QSOX2 may promote CRC cell proliferation and metastasis via some of these signaling pathways (Figure 5C). Intriguingly, cell cycle signaling pathway exhibited the most significant enrichment. Therefore, we focused on cell cycle signaling pathway for further detection. Furthermore, the expression of some cell cycle signaling pathway-related key regulators was detected by qRT-PCR. Consistent with our GEPIA analysis, the results of qRT-PCR analysis showed that QSOX2 knockdown significantly caused the accumulation of p21 and p27 in mRNA level, whereas it remarkably decreased the mRNA expression of cyclin D1, cyclin E2, CDK2, CDK4 (Figure 5D). The Western blot results showed that knockdown of QSOX2 in HCT116 and LoVo cells significantly increased the protein expression of p21 and p27, and reduced the protein expression of cyclinD1 and Notch1 (Figure 5E). More importantly, among these genes, p21 was significantly upregulated in mRNA and protein level after QSOX2 was knocked down. These results suggested that QSOX2 is very likely to promote the proliferation of colorectal cancer cells by regulating the cell cycle progression mediated by p21.

**TABLE 9:** Signaling pathways enriched in the QSOX2 high group.

Gene sets	NES	NOM *p*-value	FDR *q*-value
KEGG_CELL_CYCLE	2.435	0.000	0.000
KEGG_HOMOLOGOUS_RECOMBINATION	2.319	0.000	0.000
KEGG_PYRIMIDINE_METABOLISM	2.255	0.000	0.003
KEGG_SPLICEOSOME	2.209	0.000	0.004
KEGG_PURINE_METABOLISM	2.197	0.000	0.003
KEGG_NUCLEOTIDE_EXCISION_REPAIR	2.156	0.000	0.006
KEGG_NOTCH_SIGNALING_PATHWAY	2.029	0.006	0.010
KEGG_GLYOXYLATE_AND_ DICARBOXYLATE_METABOLISM	1.955	0.000	0.018
KEGG_UBIQUITIN_MEDIATED_ PROTEOLYSIS	1.952	0.006	0.017
KEGG_DNA_REPLICATION	1.951	0.002	0.017
KEGG_MTOR_SIGNALING_PATHWAY	1.902	0.002	0.023
KEGG_P53_SIGNALING_PATHWAY	1.856	0.020	0.032
KEGG_ERBB_SIGNALING_PATHWAY	1.855	0.006	0.031
KEGG_GLYCINE_SERINE_AND_THREONINE_METABOLISM	1.852	0.008	0.031
KEGG_BASAL_TRANSCRIPTION_FACTORS	1.829	0.016	0.037

*NES, normalized enrichment score; NOM, nominal; FDR, false discovery rate.*

### Quiescin Sulfhydryl Oxidase 2 Promotes Colorectal Cancer Cell Proliferation *in vivo*

**FIGURE 6:**
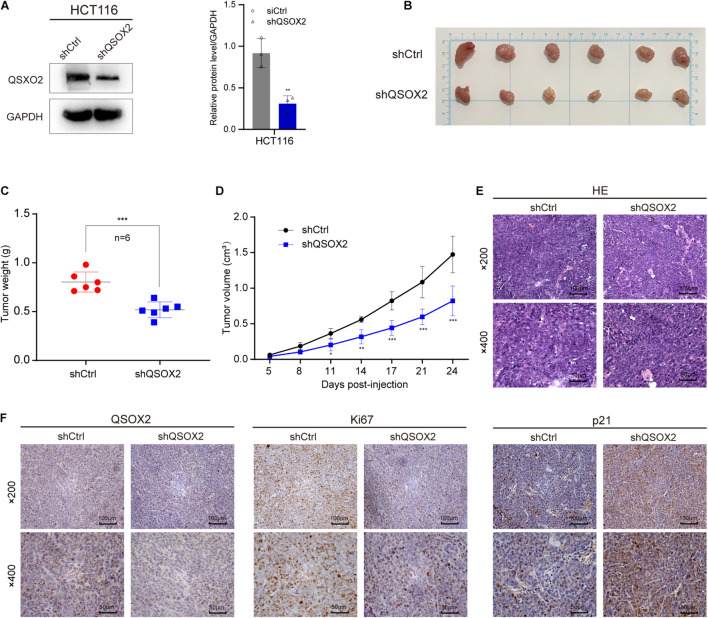
QSOX2 promotes CRC cell proliferation *in vivo*. **(A)** The protein expression of QSOX2 in HCT116 stable transfected cell line was confirmed by Western blot assay **(Left)**. Densitometry values of QSOX2 normalized to GAPDH **(Right)**. **(B)** The dissected subcutaneous tumor in mice formed by the shCtrl and shQSOX2 groups (*n* = 6 in each group). **(C)** Analysis of the weight of dissected xenograft tumors. **(D)** The growth curve of xenograft tumors in mice. **(E)** The sections of xenograft tumors were stained by H&E and IHC using QSOX2, Ki67, and p21 antibodies.

Xenograft tumorigenesis model was conducted to detect the effect of QSOX2 on proliferation of CRC cell in *in vivo*. The protein expression of QSOX2 in HCT116 stable transfected cell line was confirmed by Western blot assay (Figure 6A). The mice were sacrificed at 24 days, and the subcutaneous tumor in mice was dissected. The results showed that the xenograft tumors formed by shCtrl cells were significantly heavier and larger than that of shQSOX2 cells (Figures 6B–D). H&E staining was performed to confirm the xenograft tumors (Figure 6E). Furthermore, immunohistochemistry staining was performed to detect the expression of QSOX2, p21, and the cancer cell proliferation marker Ki67 in shQSOX2 and shCtrl groups. The staining results showed that QSOX2 stable knockdown resulted in weaker staining intensity of QSOX2 and Ki67 in the tumor sections but a stronger staining intensity of p21 (Figure 6F).

## Discussion

The increasing disease burden caused by CRC has become one of the major global threats to human health, with about 1.88 million new cases and 900,000 death cases annually (Sung et al., 2021). Although CRC has been extensively studied, more powerful and reliable biomarkers used to predict CRC progression and prognosis are still required for providing clinical significance. In the past years, with the growing development of next-generation genome sequencing technology, various data of gene expression level in CRC samples were uploaded to the public database, such as TCGA and GEO, which provides chances for biomarker discovery and validation (Long et al., 2019; Liu et al., 2020). The quiescin sulfhydryl oxidase family consists of QSOX1 and QSOX2; unlike QSOX1, which has been wildly studied in tumors, the expression and function of QSOX2 in tumors is largely unclear. With this in mind, in this study, the expression of QSOX2 in various human tumors was first analyzed via the publicly available databases TIMER2.0 and UALCAN. The results indicated that QSOX2 gene expression was upregulated in multiple solid tumors, such as colorectal cancer, bladder cancer, breast cancer, clear cell renal cell cancer, esophageal cancer, stomach cancer, and others than in their matched adjacent NTTs. Then, we focused on the expression of QSOX2 in colorectal cancer.

In the present study, we purposed to detect the expression and function of QSOX2 in CRC progression, especially its role as a prognostic biomarker. Moreover, we also tried to identify the potential signaling pathways involved in the regulation of CRC development by QSOX2. First, we evaluated the expression of QSOX2 in CRC by bioinformatic analysis based on TCGA cohort. Compared with adjacent NTTs, QSOX2 expression level was significantly upregulated in patients with CRC, and the results have been verified both in mRNA and protein level by qRT-PCR and IHC based on our paraffin-embedded and fresh frozen tissue specimen cohort. The results of meta-analysis based on GEO database are consistent with those of bioinformatics analysis and clinical trials. These results indicate that QSOX2 may act as an oncogene and play an important role in CRC initiation and progression. More specifically, QSOX2 mRNA expression was different in the clinical characteristic groups classified by pathological stage, T, N, and M stages, and these results were confirmed by frozen tissue specimen cohort. Based on this, the relationship between QSOX2 expression and clinicopathological parameters was analyzed in TCGA cohort and validated in our well-characterized clinical cohort. We demonstrated that high QSOX2 expression is closely associated with various malignant clinicopathological factors such as TNM stage, tumor diameter, lymph node metastasis, and so on. These findings may accelerate early risk recognition and precise management of CRC patients.

Prognostic biomarkers are applied to monitor postoperative treatments, evaluate the level of cancer, and predict remission or recurrence in individual patients (Nair et al., 2014). In addition, on the basis of TCGA database, Kaplan–Meier survival analysis demonstrated that the group of QSOX2 high expression exhibited poor OS than the group of QSOX2 low expression. Importantly, Cox regression analyses showed that QSOX2 expression could serve as an independent prognostic biomarker for survival in CRC patients. These results are supported by the data of clinical validation. At this point, in our research, QSOX2 was found to be not only a factor of tumor progression in CRC but also a biomarker of prognosis. The results of bioinformatics analysis and clinical verification suggest that QSOX2 overexpression was closely related to the malignant progression of CRC patients, such as the ability of proliferation and metastasis, which is also an important feature of malignant tumors. To further detect the biological function of QSOX2 in colorectal cancer cells, we performed experiments *in vitro*, such as Transwell, wounding healing, CCK-8, and EdU assays, and xenograft tumorigenesis model *in vivo*. The results showed that the proliferation and metastasis ability of HCT116 and LoVo is reduced by inhibition of QSOX2.

The QSOX2-related potential signaling pathways in CRC was analyzed by the GSEA. Of note, 15 significantly enriched signaling pathways were selected; among them, cell cycle, notch, mTOR, p53, and ERBB signaling pathway were closely related to the progression of CRC. Intriguingly, cell cycle signaling pathway exhibited the most significant enrichment. Accumulating evidence has showed that cell cycle disorders may lead to enhanced tumor cell proliferation (Otto and Sicinski, 2017). Cyclin D1, p21, and p27 were the key regulators associated with the G1 phase (Chen et al., 2019). As a well−known tumor suppressor, activation of the transcription factor p53 plays a central role in cell cycle arrest (Engeland, 2018). P53 regulates cell cycle progression by regulating some key genes, including p21 (Vogelstein et al., 2000). Correlation analysis shows that QOSX2 is negatively correlated with p21 and p27, and positively correlated with cyclin D1, but not p53, and the results of qRT-PCR showed that QSOX2 knockdown significantly caused the accumulation of p21 and p27 mRNA and the decrease in the mRNA expression of cyclin D1, cyclin E2, CDK2, CDK4, which suggests that QSOX2 may promote the proliferation of colorectal cancer by partially regulating G1/S phase transition independent of p53. The Notch signaling pathway is activated in primary colorectal cancer and plays an important part in the initiation and progression process (Tyagi et al., 2020). Notch receptors have been reported to take part in several functions of CRC cells, such as apoptosis, proliferation, angiogenesis, and cell migration (Jackstadt et al., 2019). What is also worth noticing is that the abnormal activation of Notch1 is closely related to the severity of CRC (Vinson et al., 2016). The results of GEPIA showed that QSOX2 was positively correlated with Notch1 in mRNA level, and our Western blotting results showed that knockdown of QSOX2 remarkably decreased the expression of Notch1 protein. We estimate that QSOX2 may promote the migration and proliferation by activation of Notch signaling pathway. The constituents of tumor extracellular matrix (ECM) secreted by tumor cells and non-malignant stroma are a critical factor for cancer invasion and metastasis. Since proteins in the ECM contains disulfide bonds, QSOX2, as an enzyme that generates disulfide bonds in substrate proteins, may also promote cancer cell growth, adherence, and invasion by regulating the ECM. The mechanistic target of rapamycin (mTOR) is a highly conserved kinase, and the activation of mTOR signaling pathway is closely related to CRC cell autophagy, differentiation, apoptosis, proliferation, angiogenesis, and metastasis (Wang and Zhang, 2014). Experimental and preclinical studies have proven that mTOR is an effective target for colorectal cancer therapy, and considering the close correlation between QSOX2 and mTOR, QSOX2 also may provide promising perspectives for CRC therapy.

Although our study investigated the expression and prognostic value of QSOX2 in CRC by a variety of methods and a large number of clinical samples, there are still some limitations. First, there is a lack of some specific clinical information involved in analysis, such as microsatellite instability (MSI) and surgical methods. Second, the specimens used in this study were limited to one hospital, and lack of multicenter research. Finally, the deeper molecular mechanisms of QSOX2 in CRC are not detected by experiments *in vitro* and *in vivo*. Thus, more clinical specimens and cell biology experiments are required for further investigation in the future.

In summary, based on bioinformatics analysis and clinical verification, our study demonstrated that QSOX2 is upregulated in CRC, and the upregulation of QSOX2 is closely correlated with clinical aggressive progression and poor survival in TCGA clinical and two independent specimen cohorts. We also found that QSOX2 facilitates the proliferation and metastasis ability of CRC cells, and these results suggest that QSOX2 plays a vital part in the initiation and progression of CRC. In conclusion, QSOX2 might act as a novel biomarker for prognosis prediction and a new target for biotherapy in CRC.

## Data Availability Statement

Publicly available datasets were analyzed in this study. This data can be found here: https://portal.gdc.cancer.gov/.

## Ethics Statement

The studies involving human participants were reviewed and approved by the Ethnic Committee of the Affiliated Hospital of Xuzhou Medical University. The patients/participants provided their written informed consent to participate in this study. The animal study was reviewed and approved by the Animal Care and Use Committee of Xuzhou Medical University. All standard biosecurity and institutional safety procedures have been adhered to in all the experiment procedures in this study.

## Author Contributions

TJ, JS, and RW designed the study and drafted the manuscript. LZ and XL analyzed the data, prepared the figures, and they contributed equally with TJ. HS and YX collected the surgical specimens and associated clinical data. JL assisted in the IHC staining and assessment. HW and SW helped to downloading the database from TCGA and GEO. CD and LL performed the experiments *in vitro*. All authors have read and discussed the manuscript and agreed with the final manuscript.

## Conflict of Interest

The authors declare that the research was conducted in the absence of any commercial or financial relationships that could be construed as a potential conflict of interest.

## Publisher’s Note

All claims expressed in this article are solely those of the authors and do not necessarily represent those of their affiliated organizations, or those of the publisher, the editors and the reviewers. Any product that may be evaluated in this article, or claim that may be made by its manufacturer, is not guaranteed or endorsed by the publisher.

## Footnotes

1
http://ualcan.path.uab.edu/

2
http://timer.cistrome.org/

3
http://gepia.cancer-pku.cn/

4
https://portal.gdc.cancer.gov/

## References

[B1] Araujo D. G. Nakao L. Gozzo P. Souza C. D. Balderrama V. Gugelmin E. S. (2014). Expression level of quiescin sulfhydryl oxidase 1 (QSOX1) in neuroblastomas. *Eur. J. Histochem.* 58:2228. 10.4081/ejh.2014.2228 24704990 PMC3980203

[B2] Baek J. A. Song P. H. Ko Y. Gu M. J. (2018). High expression of QSOX1 is associated with tumor invasiveness and high grades groups in prostate cancer. *Pathol. Res. Pract.* 214 964–967. 10.1016/j.prp.2018.05.019 29804717

[B3] Bai J. Wu K. Cao M. H. Yang Y. Pan Y. Liu H. (2019). SCF(FBXO22) targets HDM2 for degradation and modulates breast cancer cell invasion and metastasis. *Proc. Natl. Acad. Sci. U.S.A.* 116 11754–11763. 10.1073/pnas.1820990116 31138683 PMC6575577

[B4] Chandrashekar D. S. Bashel B. Balasubramanya S. A. H. Creighton C. J. Ponce-Rodriguez I. Chakravarthi B. (2017). UALCAN: a portal for facilitating tumor subgroup gene expression and survival analyses. *Neoplasia* 19 649–658. 10.1016/j.neo.2017.05.002 28732212 PMC5516091

[B5] Chen Y. Liu X. Wang H. Liu S. Hu N. Li X. (2019). Akt regulated phosphorylation of GSK-3beta/Cyclin D1, p21 and p27 contributes to cell proliferation through cell cycle progression from G1 to S/G2M phase in low-dose arsenite exposed HaCat cells. *Front. Pharmacol.* 10:1176. 10.3389/fphar.2019.01176 31680960 PMC6798184

[B6] Codding J. A. Israel B. A. Thorpe C. (2012). Protein substrate discrimination in the quiescin sulfhydryl oxidase (QSOX) family. *Biochemistry* 51 4226–4235. 10.1021/bi300394w 22582951 PMC3358421

[B7] Coppock D. L. Thorpe C. (2006). Multidomain flavin-dependent sulfhydryl oxidases. *Antioxid. Redox Signal.* 8 300–311. 10.1089/ars.2006.8.300 16677076

[B8] De Roock W. Claes B. Bernasconi D. De Schutter J. Biesmans B. Fountzilas G. (2010). Effects of KRAS, BRAF, NRAS, and PIK3CA mutations on the efficacy of cetuximab plus chemotherapy in chemotherapy-refractory metastatic colorectal cancer: a retrospective consortium analysis. *Lancet Oncol.* 11 753–762. 10.1016/S1470-2045(10)70130-3 20619739

[B9] Engeland K. (2018). Cell cycle arrest through indirect transcriptional repression by p53: i have a DREAM. *Cell Death Differ.* 25 114–132. 10.1038/cdd.2017.172 29125603 PMC5729532

[B10] Geng Y. Xu C. Wang Y. Zhang L. (2020). Quiescin sulfhydryl oxidase 1 regulates the proliferation, migration and invasion of human glioblastoma cells via PI3K/Akt pathway. *Onco Targets Ther.* 13 5721–5729. 10.2147/OTT.S255941 32606784 PMC7306469

[B11] Hoober K. L. Glynn N. M. Burnside J. Coppock D. L. Thorpe C. (1999). Homology between egg white sulfhydryl oxidase and quiescin Q6 defines a new class of flavin-linked sulfhydryl oxidases. *J. Biol. Chem.* 274 31759–31762. 10.1074/jbc.274.45.31759 10542195

[B12] Hou P. F. Jiang T. Chen F. Shi P. C. Li H. Q. Bai J. (2018). KIF4A facilitates cell proliferation via induction of p21-mediated cell cycle progression and promotes metastasis in colorectal cancer. *Cell Death Dis.* 9:477. 10.1038/s41419-018-0550-9 29706624 PMC5924760

[B13] Israel B. A. Jiang L. Gannon S. A. Thorpe C. (2014). Disulfide bond generation in mammalian blood serum: detection and purification of quiescin-sulfhydryl oxidase. *Free Radic. Biol. Med.* 69 129–135. 10.1016/j.freeradbiomed.2014.01.020 24468475 PMC3960832

[B14] Jackstadt R. van Hooff S. R. Leach J. D. Cortes-Lavaud X. Lohuis J. O. Ridgway R. A. (2019). Epithelial NOTCH signaling rewires the tumor microenvironment of colorectal cancer to drive poor-prognosis subtypes and metastasis. *Cancer Cell* 36 319–336.e7. 10.1016/j.ccell.2019.08.003 31526760 PMC6853173

[B15] Jiang T. Li H. Jiang R. Li H. Hou P. Chen Y. (2018). Pin2/TRF1binding protein X1 inhibits colorectal cancer cell migration and invasion in vitro and metastasis in vivo via the nuclear factorkappaB signaling pathway. *Oncol. Rep.* 40 1533–1544. 10.3892/or.2018.6570 30015978

[B16] Kamel H. F. M. Al-Amodi H. (2017). Exploitation of gene expression and cancer biomarkers in paving the path to era of personalized medicine. *Genomics Proteomics Bioinformatics* 15 220–235. 10.1016/j.gpb.2016.11.005 28813639 PMC5582794

[B17] Katchman B. A. Antwi K. Hostetter G. Demeure M. J. Watanabe A. Decker G. A. (2011). Quiescin sulfhydryl oxidase 1 promotes invasion of pancreatic tumor cells mediated by matrix metalloproteinases. *Mol. Cancer Res.* 9 1621–1631. 10.1158/1541-7786.MCR-11-0018 21989104

[B18] Knutsvik G. Collett K. Arnes J. Akslen L. A. Stefansson I. M. (2016). QSOX1 expression is associated with aggressive tumor features and reduced survival in breast carcinomas. *Mod. Pathol.* 29 1485–1491. 10.1038/modpathol.2016.148 27562495

[B19] Koncina E. Haan S. Rauh S. Letellier E. (2020). Prognostic and predictive molecular biomarkers for colorectal cancer: updates and challenges. *Cancers (Basel)* 12:319. 10.3390/cancers12020319 32019056 PMC7072488

[B20] Lacerenza S. Ciregia F. Giusti L. Bonotti A. Greco V. Giannaccini G. (2020). Putative biomarkers for malignant pleural mesothelioma suggested by proteomic analysis of cell secretome. *Cancer Genomics Proteomics* 17 225–236. 10.21873/cgp.20183 32345664 PMC7259891

[B21] Li T. Fu J. Zeng Z. Cohen D. Li J. Chen Q. (2020). TIMER2.0 for analysis of tumor-infiltrating immune cells. *Nucleic Acids Res.* 48 W509–W514. 10.1093/nar/gkaa407 32442275 PMC7319575

[B22] Liu X. Bing Z. Wu J. Zhang J. Zhou W. Ni M. (2020). Integrative gene expression profiling analysis to investigate potential prognostic biomarkers for colorectal cancer. *Med. Sci. Monit.* 26:e918906. 10.12659/MSM.918906 31893510 PMC6977628

[B23] Long N. P. Park S. Anh N. H. Nghi T. D. Yoon S. J. Park J. H. (2019). High-throughput omics and statistical learning integration for the discovery and validation of novel diagnostic signatures in colorectal cancer. *Int. J. Mol. Sci.* 20:296. 10.3390/ijms20020296 30642095 PMC6358915

[B24] Nair M. Sandhu S. S. Sharma A. K. (2014). Prognostic and predictive biomarkers in cancer. *Curr. Cancer Drug Targets* 14 477–504. 10.2174/1568009614666140506111118 24807144

[B25] Nguyen L. H. Goel A. Chung D. C. (2020). Pathways of colorectal carcinogenesis. *Gastroenterology* 158 291–302. 10.1053/j.gastro.2019.08.059 31622622 PMC6981255

[B26] Otto T. Sicinski P. (2017). Cell cycle proteins as promising targets in cancer therapy. *Nat. Rev. Cancer* 17 93–115. 10.1038/nrc.2016.138 28127048 PMC5345933

[B27] Provenzale D. Ness R. M. Llor X. Weiss J. M. Abbadessa B. Cooper G. (2020). NCCN guidelines insights: colorectal cancer screening, version 2.2020. *J. Natl. Compr. Canc. Netw.* 18 1312–1320. 10.6004/jnccn.2020.0048 33022639 PMC8311627

[B28] Sepulveda A. R. Hamilton S. R. Allegra C. J. Grody W. Cushman-Vokoun A. M. Funkhouser W. K. (2017). Molecular biomarkers for the evaluation of colorectal cancer: guideline from the American Society for Clinical Pathology, College of American Pathologists, Association for Molecular Pathology, and American Society of Clinical Oncology. *J. Mol. Diagn.* 19 187–225. 10.1016/j.jmoldx.2016.11.001 28185757 PMC5971222

[B29] Siegel R. L. Miller K. D. Fuchs H. E. Jemal A. (2021). Cancer statistics, 2021. *CA Cancer J. Clin.* 71 7–33. 10.3322/caac.21654 33433946

[B30] Subramanian A. Kuehn H. Gould J. Tamayo P. Mesirov J. P. (2007). GSEA-P: a desktop application for gene set enrichment analysis. *Bioinformatics* 23 3251–3253. 10.1093/bioinformatics/btm369 17644558

[B31] Sung H. J. Ahn J. M. Yoon Y. H. Na S. S. Choi Y. J. Kim Y. I. (2018). Quiescin sulfhydryl oxidase 1 (QSOX1) secreted by lung cancer cells promotes cancer metastasis. *Int. J. Mol. Sci.* 19:3213. 10.3390/ijms19103213 30336636 PMC6214099

[B32] Sung H. Ferlay J. Siegel R. L. Laversanne M. Soerjomataram I. Jemal A. (2021). Global cancer statistics 2020: GLOBOCAN estimates of incidence and mortality worldwide for 36 cancers in 185 countries. *CA Cancer J. Clin.* 71 209–249. 10.3322/caac.21660 33538338

[B33] Tang Z. Li C. Kang B. Gao G. Li C. Zhang Z. (2017). GEPIA: a web server for cancer and normal gene expression profiling and interactive analyses. *Nucleic Acids Res.* 45 W98–W102. 10.1093/nar/gkx247 28407145 PMC5570223

[B34] Tyagi A. Sharma A. K. Damodaran C. (2020). A review on notch signaling and colorectal cancer. *Cells* 9:1549. 10.3390/cells9061549 32630477 PMC7349609

[B35] Vinson K. E. George D. C. Fender A. W. Bertrand F. E. Sigounas G. (2016). The Notch pathway in colorectal cancer. *Int. J. Cancer* 138 1835–1842. 10.1002/ijc.29800 26264352

[B36] Vogelstein B. Lane D. Levine A. J. (2000). Surfing the p53 network. *Nature* 408 307–310. 10.1038/35042675 11099028

[B37] Wang T. E. Li S. H. Minabe S. Anderson A. L. Dun M. D. Maeda K. I. (2018). Mouse quiescin sulfhydryl oxidases exhibit distinct epididymal luminal distribution with segment-specific sperm surface associations. *Biol. Reprod.* 99 1022–1033. 10.1093/biolre/ioy125 29800099

[B38] Wang X. W. Zhang Y. J. (2014). Targeting mTOR network in colorectal cancer therapy. *World J. Gastroenterol.* 20 4178–4188. 10.3748/wjg.v20.i15.4178 24764656 PMC3989954

[B39] Zhai Z. Yu X. Yang B. Zhang Y. Zhang L. Li X. (2017). Colorectal cancer heterogeneity and targeted therapy: clinical implications, challenges and solutions for treatment resistance. *Semin. Cell Dev. Biol.* 64 107–115. 10.1016/j.semcdb.2016.08.033 27578007

