# Supplementary figures and images for: Quiescin Sulfhydryl Oxidase 2 Overexpression Predicts Poor Prognosis and Tumor Progression in Patients With Colorectal Cancer: A Study Based on Data Mining and Clinical Verification
Source: Front Cell Dev Biol. 2021 Nov 10;9:678770. doi: 10.3389/fcell.2021.678770 (PMC8631333; doi:10.3389/fcell.2021.678770)

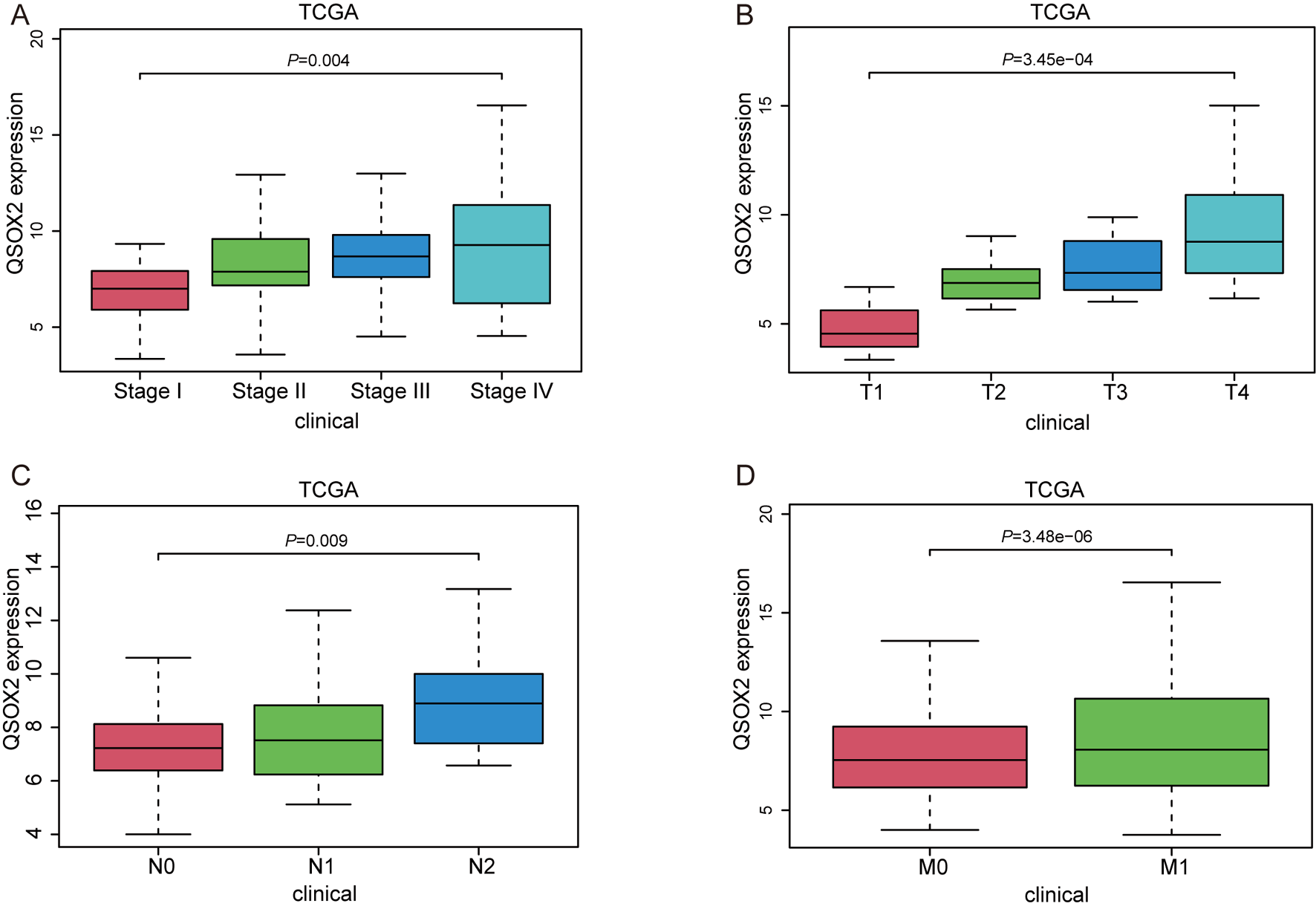

Supplement: Supplementary file 1 [file Image_1.TIF]
